# Critical Windows: Exploring the Association Between Perinatal Trauma, Epigenetics, and Chronic Pain

**DOI:** 10.1177/10738584231176233

**Published:** 2023-05-22

**Authors:** Zoe N. Kodila, Sandy R. Shultz, Glenn R. Yamakawa, Richelle Mychasiuk

**Affiliations:** 1 Department of Neuroscience, Central Clinical School, Monash University, Melbourne, Australia; 2 Health Sciences, Vancouver Island University, Nanaimo, Canada

**Keywords:** fetal programming, prenatal, neonatal, nociception, sensitivity, methylation, microRNA, intimate partner violence

## Abstract

Chronic pain is highly prevalent and burdensome, affecting millions of people worldwide. Although it emerges at any point in life, it often manifests in adolescence. Given that adolescence is a unique developmental period, additional strains associated with persistent and often idiopathic pain lead to significant long-term consequences. While there is no singular cause for the chronification of pain, epigenetic modifications that lead to neural reorganization may underpin central sensitization and subsequent manifestation of pain hypersensitivity. Epigenetic processes are particularly active during the prenatal and early postnatal years. We demonstrate how exposure to various traumas, such as intimate partner violence while in utero or adverse childhood experiences, can significantly influence epigenetic regulation within the brain and in turn modify pain-related processes. We provide compelling evidence that the burden of chronic pain is likely initiated early in life, often being transmitted from mother to offspring. We also highlight two promising prophylactic strategies, oxytocin administration and probiotic use, that have the potential to attenuate the epigenetic consequences of early adversity. Overall, we advance understanding of the causal relationship between trauma and adolescent chronic pain by highlighting epigenetic mechanisms that underlie this transmission of risk, ultimately informing how to prevent this rising epidemic.

## Introduction

Epigenetics is an evolutionarily important phenomenon that regulates neurodevelopment and neuroplasticity in response to adapting environmental conditions (Mychasiuk 2015). Hence, epigenetics modulates the interplay between DNA and environmental conditions. There has recently been a shift toward understanding the role of early life experiences in neuropathologic processes, as they may prime individuals to be more susceptible to neurologic conditions such as chronic pain. Specifically, adverse early life experiences, including those that begin in utero, can modify the epigenome and phenotypic gene expression, potentially leading to changes in protein expression, proinflammatory cytokine activity (Elwenspoek and others 2017), hypothalamic-pituitary-adrenal (HPA) axis activity (Trollope and others 2012), and mechanisms of neuroplasticity (Salinas and others 2020). Chronic pain is a highly debilitating condition that affects a significant portion of adolescents, especially females, with prevalence estimated between 11% and 38% (King and others 2011). It is normally defined as persistent pain that endures for ≥3 months (Von Korff and Dunn 2008) and commonly presents with comorbidities such as anxiety and depression (Tunks and others 2008). While symptoms can arise at any time, adolescence is recognized as a period of high risk for chronic pain onset, with symptoms often being sustained throughout an individual’s life. Therefore, the purpose of this literature review was to integrate and assess current evidence regarding the role of epigenetics and early adversity in the development of chronic pain. Specifically, we examine literature regarding the influence of intimate partner violence (IPV) in utero and early life physical neglect on the development of chronic pain during adolescence. We provide current understanding of how epigenetic and environmental factors combine to propagate the intergenerational risk for chronic pain development, potential neurophysiologic mechanisms within the central nervous system that may be underlying this transmission, and gaps within the literature (mechanistic and epidemiologic) while highlighting potential prophylactic interventions that target epigenetic mechanisms. Particularly, we demonstrate that through epigenetic programming, perinatal trauma (an often-overlooked consideration for chronic pain origin) can potentiate the development of chronic pain.

## Epigenetic Regulation of Gene Expression

Epigenetics represents the interplay between environmental factors and an individual’s genotype and involves specific molecular mechanisms that allow or inhibit gene expression in a temporal- and tissue-dependent manner (Mychasiuk 2015). These epigenetic regulations do not influence the primary nucleotide sequence but allow for adaption to selective environmental pressures, thereby conferring an immediate evolutionary advantage (Lind and Spagopoulou 2018). Epigenetic changes permit gene expression alterations between cells, which provides a foundation for cellular specialization in terms of morphology and functionality, resulting in a more adaptive organism (Mohn and Schübeler 2009; Wu and Sun 2006). Parental experiences, within the maternal and paternal lineages, significantly contribute to the offspring epigenome. Although the link between maternal experiences and child outcomes has long been established, the association between paternal experiences and epigenetic programming in offspring is a more recent discovery (Hehar and Mychasiuk 2015). Given the ongoing nature of spermatogenesis, as compared with oogenesis, the paternal environment bestows significant modulatory capacity on the offspring epigenome and subsequent development (Hehar and Mychasiuk 2015). Although not examined in the context of chronic pain, as the epigenomes from both parents combine during conception, we can infer that they are influencing risk and resiliency in this context. Given that epigenetic mechanisms can influence and regulate neuroplasticity (Mychasiuk 2015), they likely play a role in the cortical changes that underpin chronic pain neuropathology. The cell uses three key epigenetic mechanisms to regulate gene expression: DNA methylation, histone modification, and microribonucleic acids (microRNAs).

### DNA Methylation

The most frequently studied epigenetic mechanism is DNA methylation, which involves the addition of a methyl group to the C_5_ position of a cytosine nucleotide (commonly associated with a CpG island) to form a 5-methylcytosine (Moore and others 2013). DNA methylation is catalyzed by DNA methyltransferases (DNMTs), which are heavily involved in embryonic development and neuronal activity within the brain (Uysal and others 2015; Yin and others 2012). DNMTs are classified into one of three isoforms—DNMT1, DNMT3A, and DNMT3B—all of which catalyze the addition of the methyl group to the 5-carbon of the cytosine pyrimidine ring but under different contexts (Buchheit and others 2012). The addition of a methyl group to a CpG island inhibits active gene transcriptional factors from binding and results in silencing of gene expression (Denk and McMahon 2012). Alternatively, methylated CpG islands can bind to gene repressor factors such as MeCP2, which contain active repression domains resulting in suppression of gene expression (Nan and others 1997). DNMT3A and DNMT3B establish novel methylation patterns and hence are termed *de novo DNMTs*, while DNMT1 is responsible for replicating methylation patterns from parental to daughter chromosome strands during cell replication (Moore and others 2013). It is still not well understood how de novo methylation is acquired in terms of which genes are silenced and the mechanisms by which certain genes are silenced over others. One of the most popular current theories hypothesizes that the demethylation process is regulated by TET and TDG proteins. TET proteins induce or regulate the conversion of 5-methylcytosine to 5-hydroxymethylcytosine and then to 5-formylcytosine or 5-carboxylcytosine. TDG proteins are then able to excise the oxidized variations of cytosine formed by TET, at which point basic DNA repair mechanisms can restore an unmethylated cytosine nucleotide into the DNA strand (Wu and Zhang 2017). Moore and others (2013) proposed that there are multiple mechanisms for DNA methylation and demethylation that compensate for one another in varying circumstances. This suggests that DNA methylation is a vital component for adaptive gene expression as well as stable gene expression.

### Histone Modifications

Histone proteins and nucleotide sequences form nucleosomes, which are the key foundations of chromatin (Mariño-Ramírez and others 2005). There are eight core histone proteins around which nucleotide sequences can wrap. The histone proteins are composed of a core globule section and a N-terminal tail that extends outward from the core, which other proteins and epigenetic factors can interact with (Biswas and others 2011). These interactions can result in acetylation, methylation, phosphorylation, or ubiquitination modifications, commonly to the lysine residues in the tail (Bannister and Kouzarides 2011). Depending on the type of histone modification, alterations to chromatic architecture result in more compacted or loosened strands, which ultimately influences the accessibility of transcriptional factors to the coding region of the gene (Turner 2000). Histone acetylation tends to cause the tightly bound chromatic to unwind, resulting in easier access for transcription factors; this is mediated by enzymes called *histone acetyltransferases*. Histone deacetylases (HDACs) do the opposite (Yang and Seto 2007). There are 4 classes of histone deacetylation enzymes, and they all play an important role in chromatin remodeling and regulation of gene expression (Mychasiuk 2015; Seto and Yoshida 2014). HDACs can interfere with and prevent removal of acetyl groups from histone proteins. HDACs form enzymatic complexes with corepressors that are regulated through protein-protein interactions (Seto and Yoshida 2014). As their primary function is the removal of acetyl groups, they play a significant role in genetic expression by providing access to binding sites on histones for transcription promoters or repressors (Seto and Yoshida 2014).

Elevated HDAC2 expression has been linked to changes in spine density, synaptic connectivity, and memory processes, suggesting that it plays a significant role in neuroplasticity (Guan and others 2009).

### RNA Regulation

The last epigenetic mechanism of interest for this review is RNA regulation, which involves the activity of noncoding RNAs that regulate gene expression through recruitment of chromatin modulators, transcriptional repressors, and transcriptional activators or by complementary sequence binding to block translation (Mychasiuk 2015). MicroRNAs are regulatory mechanisms that usually result in gene silencing. This process involves binding of mRNA to small noncoding microRNA strands that subsequently block translation at the ribosome. MicroRNAs are capable of modifying spine morphology in hippocampal neurons (Schratt and others 2006), demonstrating that they can modulate plasticity through synaptic regulation. Similarly, noxious inflammatory stimuli induce changes in microRNA expression within the spinal cord, while knockdown of microRNA-124a increases proinflammatory markers in a mouse model (Kynast and others 2013).

## Epigenetic Regulation during Prenatal Neurodevelopment

Fetal brain development is heavily regulated by epigenetic processes that produce unique gene expression patterns leading to cell specialization and functionality. Each cell contains the same genetic material; however, epigenetically regulated expression allows for differentiation as well as neurodevelopment and neurogenesis. DNA methylation mechanisms are initiated prior to implantation, whereby there is complete demethylation of the parental genomes (Howlett and Reik 1991), followed by de novo methylation as the embryo develops (Okano and others 1999). De novo DNA methylation plays a significant role in cell formation and is a major contributor to astrocyte formation (Fan and others 2005; Takizawa and others 2001) as well as formation of glia from neural stem cells (Nakagawa and others 2020). DNA methylation also regulates expression of genes such as *Oct-4*, which code for a transcription factor that is involved in repression of cell differentiation (Hattori and others 2004). Histone modifications are necessary for fetal neurodevelopment. Studies investigating *HDAC1* activity in mouse models found that it was vital for cell differentiation through repression of growth-inhibitory factors (Lagger and others 2002; Zupkovitz and others 2006). Similarly, disruption of histone methylation via the enzyme G9a led to embryo death in a mouse model (Tachibana and others 2002; Takizawa and others 2001). There is less literature on RNA regulatory mechanisms, although small noncoding RNAs are able to cross the murine placental barrier and can be measured within the fetus; therefore, they likely influence gene expression and neurodevelopment (Li and others 2015). Finally, epigenetics likely contributes to the development of sex differences, which begin later in fetal development (Scott and others 2011). While there are still many unknowns, epigenetic regulation is pivotal for development of the CNS and cell proliferation. As such, environmental factors associated with maternal health may lead to alterations within the fetal epigenome and subsequent development, which prime the body for the manifestation of chronic pain during postnatal life.

### Fetal Programming, Epigenetics, and the Maternal Environment

**Figure 1.**
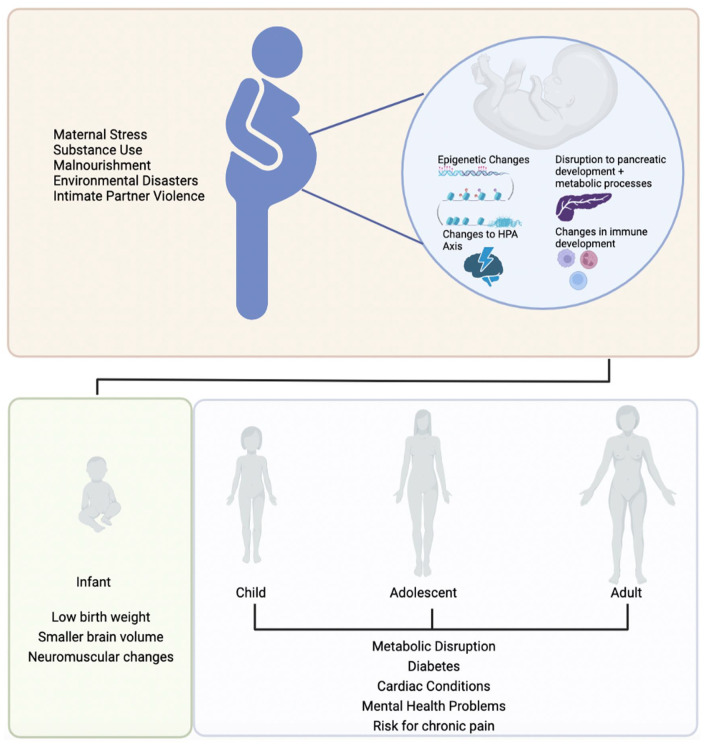
Maternal factors and the timeline whereby they lead to health complications in subsequent generations. As there are various maternal factors capable of modifying fetal epigenetic profiles, a known mismatch between prenatal and postnatal environment influences many health benefits or complications during adolescence and adulthood. HPA = hypothalamic-pituitary-adrenal.

As epigenetic regulation is highly prevalent during prenatal development, the intrauterine environment is incredibly important for establishing the fetal epigenome. The interplay between the epigenome and the intrauterine environment is known as *fetal programming*, whereby maternal factors influence developmental trajectories of the offspring by priming the fetus for certain environmental conditions (Hocher 2014). Fetal programming is an evolutionarily beneficial process as it allows for rapid adaptation, opposed to slower-acting mendelian inheritance. Fetal programming represents a situation where the fetus “gambles” that its future environment will resemble its in utero experience. However, in cases where there is a mismatch between the in utero environment and the conditions of postnatal development, this gamble can result in health complications and a predisposition for many noncommunicable illnesses, such as coronary heart disease, liver problems, and neuropsychiatric disorders (Godfrey and Barker 2001; Figure 1).

The majority of fetal programming research has been conducted within the context of maternal malnourishment and insulin resistance in offspring (Hocher 2014). These studies have demonstrated that maternal malnourishment during pregnancy alters offspring gene expression, which leads to alterations in metabolic homeostasis that prepare the fetus for caloric deficits during postnatal life (Gicquel and others 2008). However, when there is a surplus of calories during infancy and adolescence and into adulthood, these fetal-induced alterations to the metabolic phenotype can lead to insulin resistance and diabetes (Zhu and others 2019). More recently, scientific literature has explored maternal stress and its influence on fetal programming and the epigenome (Mychasiuk and others 2011). Li and others (2012) showed that maternal cortisol levels were inversely proportional to the growth of the fetal brain. Moreover, current literature indicates that elevated maternal cortisol can influence the development of the HPA axis during gestation (Irwin and others 2021). As maternal cortisol crosses the placental barrier and reaches the developing fetus, the placenta has evolved to prevent excess maternal cortisol from influencing development. The enzyme 11β-HSD2 (11β-hydroxysteroid dehydrogenase type 2) oxidizes cortisol to its biologically inactive form cortisone to prevent it from negatively interacting with the developing fetus (Brown and others 1996). Yet, in cases of elevated maternal cortisol levels, enzymatic activity in the placenta is saturated, which results in active cortisol flooding the intrauterine environment. These cortisol levels are often toxic to the developing fetus, altering development of the fetal brain. Specifically, excessive cortisol can modulate maturation and migration of inhibitory GABAergic neurons, which are important for establishing the cortical structure during fetal neurodevelopment (Fine and others 2014). Additionally, maternal anxiety and proinflammatory cytokine activity may down-regulate 11β-HSD2 activity (Kossintseva and others 2006; O’Donnell and others 2012), further increasing the levels of maternal cortisol that cross the placenta.

Moreover, studies have demonstrated that elevated maternal immune activity may alter epigenetic mechanisms (Basil and others 2014; Han and others 2021; Labouesse and others 2015). For example, maternal immune activation was able to induce hypomethylation of the *MeCP2* gene within the hypothalamus in adolescent mice (Basil and others 2014). Changes in *MeCP2* activity have been linked to changes in dopaminergic neuronal branching and neural connectivity (Gantz and others 2011). Reduced activity of dopaminergic neurons has been associated with reduced analgesic effects (Wood and others 2007), although demonstrating that these three mechanisms drive the chronification of pain has not transpired to date. In addition, maternal immune activity has been linked to reduced expression of *GAD1* and *GAD2* in the prefrontal cortex (PFC) of offspring in a murine model (Labouesse and others 2015). As such, it is evident that maternal factors can produce changes in offspring health by modulating epigenetic activity.

It is important to note that maternal influences on the epigenome and subsequent neurodevelopment of offspring likely occur in a sex-dependent manner (Bale 2011), which may underpin the physiologic differences associated with higher rates of chronic pain in females (Mychasiuk and others 2011). Elevated levels of maternal and placental cortisol during pregnancy influence male and female fetuses differently (Sandman and others 2013). In males but not females, neuromuscular and cognitive abnormalities are often observed throughout gestation and postnatal year 1 (Sandman and others 2013). Temperamental and affective abnormalities were observed in females who were exposed to elevated maternal cortisol at 2 years of age, and reduced brain volume was observed in female youth aged 6 to 9 years (Buss and others 2011). Additionally, elevated maternal cortisol enlarged the right amygdala in females at ~7 years of age but not in males (Buss and others 2012). While this study did not investigate these parameters in relation to chronic pain, changes in brain anatomy, cognition, and affective behaviors are hallmark features of chronic pain, suggesting that fetal programming may be related to a predisposition to chronic pain in a sex-dependent manner. As such, maternal health influences the intrauterine environment and neurodevelopment and so should be considered a risk factor for the development of chronic pain.

### IPV during Pregnancy

IPV is an unfortunately common form of abuse that affects women daily on a global scale (Chibber and Krishnan 2011; Mojahed and others 2021) and frequently arises or escalates in severity during pregnancy (Campo 2015). Globally, ~27% of women aged 15 to 49 years have experienced physical or sexual abuse or both from their partners (Sardinha and others 2022), and based on an American study, 6.1% of women were abused during pregnancy (Martin and others 2001). Given that many women never report their abuse or inform authorities, this statistic is likely a conservative estimate (Gracia 2004). The literature regarding the health of women who have experienced IPV is scarce and even lacking for women who were pregnant at the time of IPV. This is partially due to the nature of IPV, as women often endanger themselves by indulging this information or participating in research (Hellmuth and Leonard 2013). As such, animal models are necessary to clarify neuropathologic and behavioral outcomes related to IPV. However, there are currently very few robust and clinically relevant preclinical models, especially when considering the scale of the problem. There are also problems with existing definitions of IPV and violence against women. The definition has purposefully been kept broad and includes financial, physical, and sexual abuse, as well as emotional manipulation, coercion, and threats against women (United Nations 1993). This broad definition highlights the nuance of the issue, as well as the scale of gender-based violence that can occur in public or private, but it results in incomplete data regarding the specific consequences of physical abuse (Ruiz-Pérez and others 2007). All forms of abuse can have severe long-term consequences, yet for clarity of this review, we focus primarily on physical abuse in pregnant women.

Pregnancy involves significant physiologic and anatomic changes to the women’s body: elevated cardiac output, decreased oxygen reserves, elevated stress hormones, and other metabolic changes that are required to meet the demands of a developing fetus (Tan and Tan 2013). The added health complications associated with physical abuse can put the mother and fetus in physiologically dangerous situations (Donovan and others 2016). The physical abuse that pregnant women endure can vary quite significantly in terms of the type of abuse and the severity. However, head and neck injuries are the most common forms of physical abuse sustained, with 40% of participants who experienced physical abuse presenting injuries in these regions (Bhandari and others 2006). Physical abuse often involves trauma to the brain via direct impact to the skull and/or through hypoxia-induced strangulation (Monahan and others 2022). These injuries are rarely sustained in isolation, with most women experiencing strangulation in addition to multiple types of blunt force trauma (Shields and others 2010). Women who have sustained a traumatic brain injury are more likely to have long-term CNS complications as compared with abused women who do not sustain a traumatic brain injury (Campbell and others 2018). As such, IPV is a large-scale tragedy that affects the lives of millions of women and may in turn affect subsequent generations through fetal programming.

**Figure 2.**
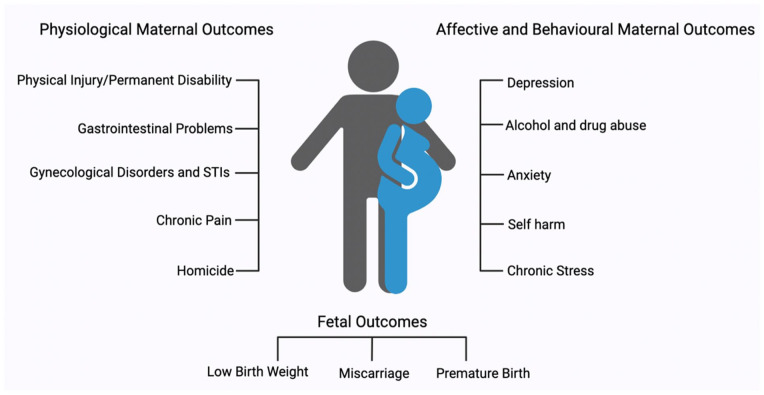
Overview of basic health complications associated with intimate partner violence. STI = sexually transmitted infection.

The majority of adverse outcomes investigated in infants exposed to IPV while in utero have focused on short-term outcomes and include low birth weight, preterm labor, and perinatal death (Donovan and others 2016; Figure 2). To our knowledge, only one study has investigated the influence of traumatic brain injury during pregnancy, which established increased depressive behaviors in the offspring. Beyond this, there is very limited information (Saber and others 2021)

Women who experience IPV during pregnancy are three times more likely to have perinatal death than pregnant women who do not experience IPV, although this can be partially rectified through IPV screening at pregnancy clinics and inclusion of appropriate medical support throughout pregnancy (Pastor-Moreno and others 2020). Although there is an abundance of research on fetal programming linked to prenatal stress, such as neurologic changes, mental illness, inflammatory factors, and increased rates of substance use, to our knowledge only one study has examined these outcomes in the context of prenatal IPV, which determined that such infants are at an increased risk of neurologic delay (Udo and others 2016). The long-term implications of intergenerational trauma have been established in the offspring of genocide survivors, war veterans, and famine sufferers (Matz and others 2015; Yehuda and Lehrner 2018). However, an important gap in the literature still exists within the context of noncortisol-induced endocrine and neuroplastic changes in the developing fetus. For example, Radtke and others (2011) found increased methylation of the glucocorticoid receptor (*GR*) in the offspring of victims of IPV, although this study included a broad definition of abuse that was not limited to physical. Unfortunately, Radtke’s study is the only identified literature investigating epigenetic changes in the offspring of IPV victims. Changes in *GR* expression may alter HPA activity and produce changes in the stress response of the offspring later in life (Meaney and others 2007; Mychasiuk and others 2011). Based on current literature demonstrating that the intrauterine environment is important for influencing neurodevelopment, it is worth assessing IPV as a potential contributor to epigenetic regulation of gene expression and subsequent chronic pain in offspring.

## Epigenetics and Early Life Neurodevelopment during Infancy

The human brain continues to mature through childhood, adolescence, and early adulthood, and epigenetic mechanisms influence these developmental processes. The first few years of life represent a period of rapid growth in myelination, functional connectivity, and cortical gray matter development (Gilmore and others 2018; Knickmeyer and others 2008). Sexual dimorphisms also become more apparent during early development with noted differences in regional volumetric measures. For example, Knickmeyer and others (2014) found that females have larger dorsolateral PFCs, a region implicated in chronic pain development. While prenatal brain development is characterized largely by proliferation, migration, and specialization, postnatal/early childhood neurodevelopment is largely driven by synaptogenesis and synaptic pruning, resulting in more efficient and plastic neural networks (Tierney and Nelson 2009). The generation of efficient neural networks involves activity-dependent synaptic strengthening referred to as *long-term potentiation* (Madison and others 1991). A study conducted in *Aplysia* demonstrated that histone acetylation and chromatic remodeling are involved in synaptic plasticity (Guan and others 2002), suggesting that they are relevant mechanisms for long-term potentiation throughout childhood. Other relevant genes involved in neuroplastic processes, such as dendritic branching and neural connectivity, include brain-derived neurotrophic factor (*BDNF*; Bathina and Das 2015), which requires phosphorylation of *MeCP2* to drive up-regulated expression and consequently increased branching within a mouse model (Zhou and others 2006). As such, epigenetic mechanisms dictate neural connectivity and may mediate neural network changes that predispose individuals to heightened pain sensitivity during adolescence and adulthood, including changes in mesocorticolimbic communication. In Table 1 we provide a brief summary of selected genes that may undergo epigenetic modulation following perinatal trauma and subsequently lead to increased risk for the development of chronic pain. Although more research is needed to identify additional genes of interest and determine if there is a causal relationship between them and chronic pain, their ability to act across circumstances highlights their potential importance and need for further investigation.

**Table 1.** Selection of Genes That May Be Involved in the Epigenetic Transmission of Risk for Chronic Pain Following Pre- and Postnatal Trauma and Onset of Chronic Pain.

Gene Name	Primary Function	Role in Prenatal Trauma	Role in ACEs	Role in Chronic Pain
*BDNF* (brain-derived neurotrophic factor)	Neurotrophic growth factor involved in development and neuroplasticity (Miranda and others 2019)	BDNF was reduced in pregnant females with PTSD and depression (Yang and others 2016). Limited evidence on IPV or specific mechanisms of epigenetic regulation.	Preclinical model of early life maltreatment persistently increased methylation of the *BDNF* exon IX (Roth and others 2009).	BDNF minimally involved in acute pain but prominently in the chronification of pain in a *BDNF* knockout mouse (Sikandar and others 2018).
*NR3C1/GR*	Encodes the glucocorticoid receptor; role in HPA axis activity (NCBI 2022).	Maternal prenatal stress increased methylation of *NR3C1* and increased expression of *NR3C1* within the placenta (Capron and others 2018). Meta-analysis demonstrated relationship between maternal stress and methylation profiles of *NR3C1* in offspring (Palma-Gudiel and others 2015).	Altered methylation of *NR3C1* gene in survivors of childhood abuse (McGowan and others 2009). Childhood trauma, including physical neglect, increased methylation of *NR3C1* (Perroud and others 2011).	Increased methylation of *NRC31* in a rodent model of chronic stress–related pain hypersensitivity (Hong and others 2015).
*MeCP2*	Involved in chromatin remodeling (MedlinePlus Genetics 2022).	Increased maternal immune activity in mice reduced methylation levels of the *MeCP2* gene (Basil and others 2014).	None	Increased expression of *MeCP2* in a model of intergenerational chronic pain (Tao and others 2020).
*TNF-a*	Encodes a proinflammatory cytokine (PubChem 2022b).	Prenatal stress increased *TNF-a* expression in the frontal cortex of offspring (Szczesny and others 2014).	Children with a history of trauma exhibit increased expression of *TNF-a* (Bücker and others 2015).	TNF-a increased in female rats following chronic constriction injury of the sciatic nerve (Schäfers and others 2003). Increased TNF-a levels in the hippocampus postinjury in male rats (Ignatowski and others 1999).
Dopaminergic system encompassing *DRD2*, *COMT*, and *MAOA*	Regulates neurotransmission important in mediating reward and motivation responses.	Prenatal stress increased *DR-2* receptor expression (Berger and others 2002).	Maternal separation decreased dopamine receptors in the PFC (Rentesi and others 2013). In mice, no association between *MAOA* methylation in the brain and early life adversity (Mattern and others 2019).	DNA methylation increased *COMT* in patients with fibromyalgia and chronic fatigue (Polli and others 2022).
*FKBP51*	Prominent role in regulation of the HPA axis, glucose tolerance (Hähle and others 2019), and protein trafficking (PubChem 2022a).	Prenatal stress resulted in persistent hypermethylation of three regions of *FKBP51* in the left dorsal hippocampus (Plank and others 2021).	Physical abuse resulted in hypomethylation of the *FKBP51* gene that was dependent on individual allele type (Klengel and others 2013).	No current evidence of epigenetic regulation of *FKBP51* in chronic pain, although it does play a role (Maiarù and others 2016).

ACE = adverse childhood experience; HPA = hypothalamic-pituitary-adrenal; IPV = intimate partner violence; PFC = prefrontal cortex; PTSD = posttraumatic stress disorder.

### Adverse Childhood Experiences

Adverse childhood experiences (ACEs) are potentially traumatic experiences that can have lifelong influences on psychological, social, and physical well-being (Boullier and Blair 2018). ACEs have been implicated in a variety of diseases and negative health outcomes (Felitti and others 1998; Kalmakis and Chandler 2015). Chronic pain is more prevalent in individuals exposed to ACEs as compared with the general population (Groenewald and others 2020). Furthermore, current trends suggest that women experience ACEs at a higher rate than males (Haahr-Pedersen and others 2020), which may correspond to the sex differences noted in patients with chronic pain (King and others 2011). Similar to patterns of IPV, ACEs tend to be more common in populations characterized by lower socioeconomic status and financial instability (Walsh and others 2019). As implied by their definition, ACEs occur during early childhood, which is when the PFC, amygdala, and hippocampus are still particularly plastic and sensitive to external influences. As a result, ACEs exercise great influence over development of the CNS and formation of neural networks.

ACEs are commonly assessed and quantified with the ACE questionnaire (World Health Organization 2020), which focuses on childhood experiences related to maltreatment (physical or sexual abuse, physical neglect) or household dysfunction (being witness to violence, drugs, or prison time of a relative; Boullier and Blair 2018). More recent literature has highlighted the impact of social and economic traumatic events that occur during childhood, such as poverty, peer victimization, and bullying, as these early experiences have demonstrated an influence on long-term physical and mental health (Finkelhor and others 2015).

**Figure 3.**
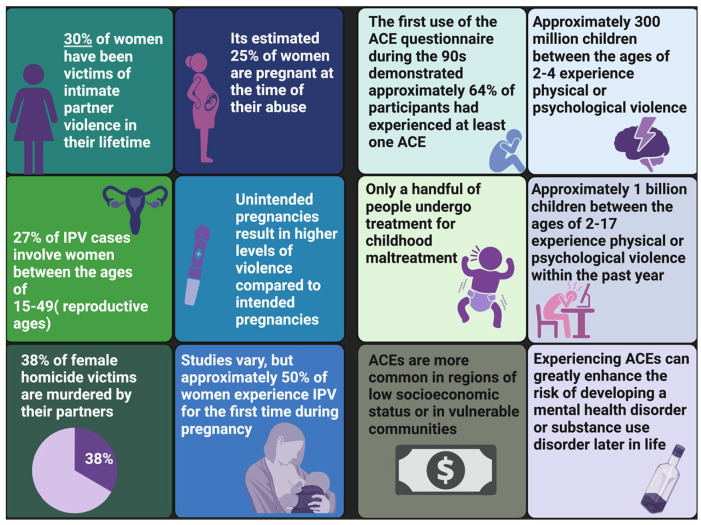
An infographic detailing statistics relevant for IPV (García-Moreno and others 2005; World Health Organization 2021) and ACEs (Boullier and Blair 2018; World Health Organization 2022) based on data collected from the World Health Organization. ACE = adverse childhood experience; IPV = intimate partner violence.

Physical neglect is an ACE characterized by failure of the parents or caregivers to properly care for their children, including failing to feed, clothe, protect, or manage their health requirements (Boullier and Blair 2018). The ACE of physical neglect is also associated with the development of chronic pain later in life (Davis and others 2005). Importantly, physical neglect can be recapitulated in preclinical studies with the use of the maternal separation paradigm, whereby pups are removed and isolated from their mother during the first 2 weeks of development (Vetulani 2013). This is a critical period of brain development, wherein sensory and nociceptive pathways are still maturing (Bâ and Seri 1993). Preclinical studies demonstrate that maternal separation alters development of the HPA axis (Aisa and others 2007) and nociception. Maternal separation has been associated with increased H3 histone acetylation and decreased methylation within the promoter region of the corticotrophin-releasing hormone gene (*CRH*; Wang and others 2014). Although this study did not examine nociceptive outcomes, it did show that early life trauma influences neuroendocrine functions and gene expression within the CNS. Similar findings have been replicated in human childhood abuse survivors who exhibit decreased levels of glucocorticoid receptor mRNA due to methylation changes on the *NR3C1* gene, when quantified in adulthood (McGowan and others 2009). Glucocorticoid receptors have also been implicated in mouse models, with changes in acetylation being linked to maternal behaviors such as grooming and arched-back nursing (Weaver and others 2004). Clinical research has demonstrated that neglect can induce increased activity within the amygdala and lead to changes in a child’s ability to register affective and social cues (Tottenham and others 2011). Increased amygdala activity has also been noted in models of chronic pain (Neugebauer and others 2004). As such, changes in neuronal activity within the amygdala after physical neglect induce functional and structural transitions that may underpin the development of persistent pain. However, it is important to note that many of the negative effects associated with ACEs are ameliorated when resiliency factors are present. Particularly in the context of intergenerational transmission of chronic pain, studies have shown that positive coping strategies, supportive social groups, and elevated socioeconomic status can buffer the negative consequences of ACEs (Beveridge and others 2022; Chang and others 2022; Nimbley and others 2023). Therefore, although early life experiences do modify development of the neural circuitry related to persistent pain via epigenetic regulation of gene expression, this does not always occur in a negative fashion. In Figure 3, we highlight a subset of the consequences associated with IPV and ACEs.

## An Overview of Chronic Pain and the Underlying Neuropathology

### Prevalence and Epidemiology in Adolescence

Chronic pain is a complex and multidimensional disease characterized by nociceptive, cognitive, affective, and behavioral changes. It is recognized as one of the most burdensome diseases globally and represents a significant disruption in quality of life (Blyth and others 2001). While the use of animal modeling precludes our ability to understand the subjective and affective components of pain, it does allow for targeted investigations into neurologic mechanisms that drive changes in nociception and the transition and manifestation of chronic pain pathology. Pain in rodents is typically assessed through observation of paw withdrawal following exposure to a mechanical or thermal stimulus. As such, preclinical assessment of pain is primarily dependent on nociceptive responsivity and does not often recapitulate the emotional and experience-dependent components of human-specific pain. Therefore, while rodent pain models lack components of persistent pain symptomology typically observed clinical populations, they do allow for invasive neurophysiologic assessment of the mechanistic processes involved in epigenetically derived nociceptive changes. Chronic pain is generally defined as a form of persistent pain that lasts for 3 months, as measured over a 6-month period (Treede and others 2015). It is also characterized by the experience of allodynia, the experience of pain in response to an innocuous stimulus, and/or hyperalgesia, the experience of an exacerbated pain response following exposure to a noxious stimulus (Jensen and Finnerup 2014). Although chronic pain may arise in response to an initial injury, it is frequently idiopathic, presenting with no defined origin.

Although some factors may increase the likelihood of developing chronic pain, it is a heterogenous and complicated disease that typically manifests in adolescence. Alarmingly, the development of chronic pain during adolescence often predisposes an individual to chronic pain throughout adulthood, which has significant implications for overall quality of life (Hadi and others 2019). The PFC is one of the brain regions with the greatest capacity for neuroplastic change during adolescence. Sensory and motor neural cortices tend to develop earlier, while regions that are involved in cognitive, social, and judgment-based decisions develop later (e.g., the PFC; Arain and others 2013). The functional and physiologic mechanisms with the greatest plasticity in the adolescent brain likely confer evolutionary benefit by allowing them to engage more readily and independently with their environment. However, this sensitivity to environmental factors increases risk and susceptibility to a plethora of illnesses, such as depression and anxiety (Kessler and others 2007; Paus and others 2008). As such, adolescence represents a significant window of sensitivity for which an individual is susceptible to positive and negative experiences.

### Mesocorticolimbic System in Chronic Pain

The mesocorticolimbic system includes several functionally distinct regions—such as the PFC, amygdala, nucleus accumbens (NAc), ventral tegmentum, and hippocampus—and is implicated in pain as well functions related to higher-order thinking, memory, and affective response (Vachon-Presseau and others 2016). The amygdala and PFC are involved in the acute pain response, as they integrate emotional information and incoming afferent information regarding painful stimulus (Ong and others 2019). Interestingly, in a human study assessing changes in gray matter volume with voxel-based morphometry, individuals with chronic lower back pain exhibited global decreases in gray matter volume that exceeded age-expected changes, with noticeable decreases in the dorsolateral PFC (Apkarian and others 2004). However, although patients were age and sex matched, the authors did not disclose whether the participants were undergoing pharmacologic treatment, which may have influenced the brain changes that they identified. That being said, numerous studies investigating patients with migraine (Schmidt-Wilcke and others 2005), complex regional pain disorder (Geha and others 2008), and fibromyalgia (Kuchinad and others 2007) also identified changes in gray matter volume. Although the mechanisms underlying the changes to gray matter cannot be elucidated in clinical cohorts, rodent studies demonstrate that these changes may be due to alterations in dendritic morphology (Kang and others 2019), thereby suggesting that chronic pain reorganizes gray matter architecture within the brain. Moreover, glutamate levels in the PFC of rodents and humans with chronic pain are reduced, indicating that excitatory signaling is distorted, which may subsequently result in negative emotional and cognitive outcomes (Kelly and others 2016; Naylor and others 2019). Similarly, activation of GABAergic inhibitory neurons within the prelimbic region, a substructure of the rodent PFC that is similar to the ventromedial PFC in terms of function, is significantly reduced in models of pain (Zhang and others 2015). Taken together, these findings suggest that chronic pain is associated with modulation to excitatory and inhibitory neurotransmission within the PFC.

As the PFC of adolescents is undergoing significant maturation, modulation at the neurotransmitter level and/or neural connectivity can happen quite readily and has the potential to induce persistent, long-term changes. In alignment with this, changes to the connectivity and communication among the PFC, amygdala, and NAc may be important for the transition from acute to chronic pain (Baliki and others 2011). Chronic pain includes its own centrally driven pain response that differs mechanistically from the acute pain response. Patients with chronic lower back pain demonstrated increased white matter connectivity among the PFC, amygdala, and NAc over a 3-year testing period (Baliki and others 2012). It has been suggested that the functional expansion between the NAc and PFC results from an increase in emotional salience, although how this relates to the chronification of pain is less well understood (Vachon-Presseau and others 2016). Increased activity within the PFC, amygdala, and NAc may result in amplification of a pain responses via integration of nociceptive information with emotional salience (Vachon-Presseau and others 2016). Alternatively, these changes in connectivity may interfere with dopaminergic signaling and other neurotransmitter activity, resulting in altered synaptic and neural communications, reorganization of neural networks, and distorted nociceptive inputs.

Evidence from fMRI data has implicated the PFC in chronic pain. Interhemispheric connectivity between the left and right dorsolateral PFC was important in analgesia and intensity of pain responses (Sevel and others 2016), while a study by Youssef and others (2014) demonstrated that cerebral blood flow increased within the dorsolateral PFC of patients with chronic pain. Increased cerebral blood flow may be indicative of increased activity in the dorsolateral PFC, suggesting that this brain region is attempting to control or suppress the ongoing nociceptive input. Alternatively, the activity in the dorsolateral PFC may be modulating behavioral and affective outcomes, such as those associated with catastrophizing beliefs, which are common in individuals with chronic pain (Gracely and others 2004). Unfortunately, while it is well understood that modifications to the corticolimbic and mesocorticolimbic systems underpin chronic pain pathology, how they translate into altered nociceptive input is still poorly understood. Furthermore, additional investigation is necessary into the underlying mechanisms that regulate changes in plasticity and neural connectivity to produce large-scale cortical reorganization observed in patients with chronic pain.

## A Complicated Relationship: Epigenetics, Trauma, and the Development of Chronic Pain

Current literature demonstrates that trauma influences epigenetic processes that may predispose individuals to chronic health conditions. Although our existing knowledge of the epigenetic mechanisms driving changes in chronic pain risk is still emerging, it is becoming clear that early life experiences play a significant role. In what follows, we highlight a few studies that have investigated the role of epigenetics on chronic pain.

**Figure 4.**
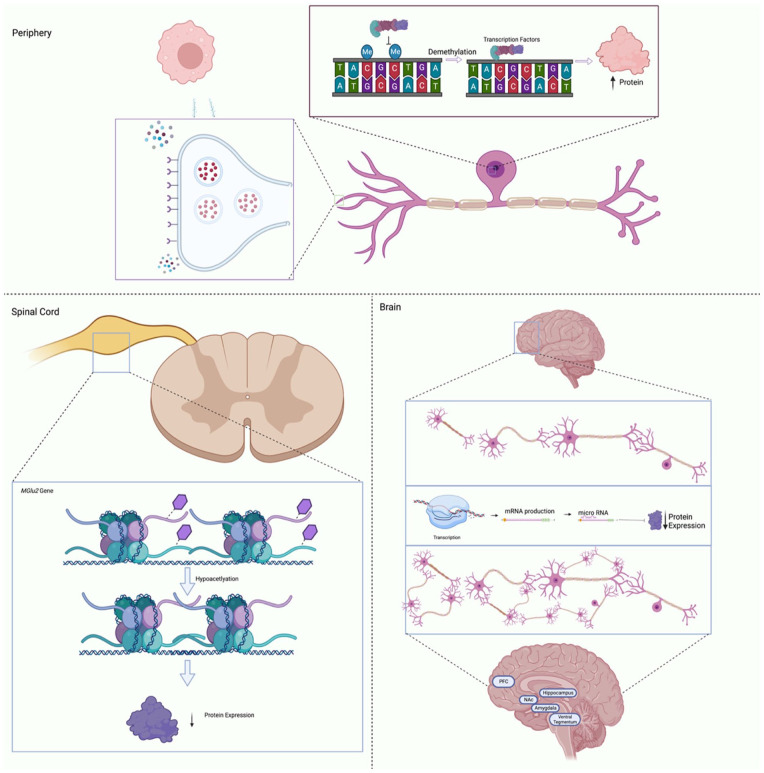
Simplified schematic detailing the basic processes whereby epigenetic regulatory factors may influence gene expression and chronic pain development. Within peripheral nerves (top panel), changes to methylation levels may increase protein expression, resulting in increased receptor density in nociceptors or cytokine secretion by immune cells. In the dorsal root ganglion of the spinal cord (left panel), histone deacetylation induced through physiologic changes can lead to increased pain sensations. Additionally, RNA regulation within the brain (right panel) may inhibit expression of dendritic inhibitory factors resulting in increased branching among the prefrontal cortex (PFC), amygdala, and nucleus accumbens (NAc).

DNA methylation studies have demonstrated changes in the methylome following neuropathic injury at chronic time points (Massart and others 2016; Tajerian and others 2013). These studies have identified chronic alterations in DNA methylation patterns systemically with changes present in T cells (Massart and others 2016) and within the spinal cord (Tajerian and others 2013), suggesting that DNA methylation aids in peripheral and central sensitization. For example, methylation changes were observed in opioid, glutamate, and dopamine receptor genes, all of which have been linked to modifications in pain thresholds (Massart and others 2016). Alterations in methylation profiles have also been highlighted in human populations with higher exposure to early life adversity (Wiegand and others 2021). However, whether these changes are contributing to changes in nociception is still poorly understood, and we can therefore only infer correlational associations and not causal relationships. Additionally, Garriga and others (2018) reported that methylation patterns differ in a temporal- and regional-dependent manner following spinal nerve ligation in rodents. At an acute time point, methylation levels increased, yet at subacute and chronic time points, methylation within the dorsal root ganglion (DRG) decreased while hypermethylation was observed in spinal cord and PFC (Garriga and others 2018). These studies showed that although methylation patterns change across time and region of interest, they may contribute to changes in CNS pain sensitivity. See Figure 4 for potential mechanisms of epigenetic regulation of chronic pain development and sensitization. Furthermore, a rodent study demonstrated that methylation patterns undergo remodeling in the peripheral nervous system and DRG following nerve injury (Gölzenleuchter and others 2015), corroborating the hypotheses that epigenetic processes that may drive persistent pain phenotypes. The DRG is a key relay station for nociceptive information between the peripheral and central nervous systems and, as such, plays a substantial role in the chronification of pain. For example, hyperexcitation in the DRG significantly contributes to sensitization in neuropathic pain, and while the specific mechanisms are still under investigation, animal models suggest that up-regulation of *DNMT* gene expression occurs within the DRG following spared nerve injury (Pollema-Mays and others 2014).

Additionally, DNA methylation contributes to the systemic aspects of chronic pain by influencing expression of proinflammatory cytokine levels. For example, methylation patterns were examined in patients with short- and long-term persistent lower back pain (6 weeks and 6 months). Individuals with long-term persistent pain had lower overall levels of DNA methylation and higher levels of mRNA expression for proinflammatory cytokines such as IL-2 (Eller and others 2021). Similarly, chronic nonspecific lower back pain was associated with changes in overall methylation patterns and hypomethylation of immune-related genes (Aroke and others 2020). Taken together, these results suggest that DNA methylation may influence the development of inflammatory and neuropathic-related pathology in chronic pain conditions. Surprisingly, a meta-analysis investigating the relationship between early life trauma and circulating markers of inflammation in adolescents concluded that associations between early life adversity and inflammatory markers were quite small (Kuhlman and others 2020). However, the study was limited in that it assessed only two inflammatory markers (IL-6 and C-reactive protein), of which C-reactive protein alone demonstrated significant change. As such, it is currently challenging to generate robust conclusions regarding the mechanisms driving the associations among inflammation, early life trauma, and chronic pain as there is a scarcity of literature.

Additionally, Hobo and others (2011) found that the administration of an HDAC inhibitor, valproate, increased expression of multiple glutamate transporters within the spinal dorsal horn, leading to decreased sensitivity following spinal nerve ligation. Central sensitization of the spinal cord and increased long-term potentiation are important steps in hypersensitive responses to noxious or, in some cases, innocuous stimuli (Ji and others 2003). As such, acetylation patterns, HDACs, and HDAC inhibitors likely play important roles in chronic pain–associated neuroplasticity (Levenson and others 2004). HDACs have also been linked to changes in expression of glutamic acid decarboxylase (GAD65), an enzyme necessary for the synthesis of GABA. Administration of HDAC inhibitors to wild type mice induced an increase in GAD65 expression, improved GABA synaptic functions, and resulted in mice with higher baseline thresholds of pain (Zhang and others 2011). Similar to other epigenetic processes, histone modifications independently regulate each gene separately, in a spatially and temporally independent manner. Beyond neuroplasticity, histone acetylation patterns modulate immune factors to prolong the inflammation often associated with chronic pain. Inflammatory mediators such as Nf-kB are heavily regulated via epigenetic mechanisms; in turn, proinflammatory mediators such as IL-1β (Ito and others 2000) and TNF-α (Rahman and others 2002) promote hyperacetylation of inflammatory promoter regions. As the immune system plays a significant role in pain sensitization whereby immune factors exacerbate hyperexcitability in nociceptors (Marchand and others 2005), it is possible that histone modifications are involved in sensitization processes in the peripheral and central nervous systems via regulation of neuroimmune properties. However, current understanding of histone modifications in the transition of acute to chronic pain is poorly understood, and further research is needed to establish the specific mechanisms driving this relationship. Based on their larger roles in synaptic regulation and cortical development, histone modifications may be regulating cortical reorganization within the CNS following exposure to perintatal trauma, thereby driving subsequent chronic pain development.

Finally, there is evidence that RNA regulation and microRNAs modulate the transition from acute to chronic pain in the context of perinatal trauma. Unique microRNA profiles have been identified in victims of childhood trauma with specific changes in expression of let-7g-5p, miR-103a-3p, miR-107, and miR-142-3p, which are associated with neurodevelopment and depression (Van der Auwera 2019). However, this study did not assess nociceptive outcomes. Although these changes in microRNA profiles are relevant to chronic pain development and early life trauma, they were not examined in a manner that would permit associative comparisons.

In addition to early life trauma, patients with persistent migraines exhibit altered microRNA profiles, including up-regulation in miR-27B and down-regulation in expression of miR-181a, let-7B, and miR-22 (Tafuri and others 2015), suggesting that pathologic pain may involve unique expression of regulatory RNA. MicroRNA changes within cerebrospinal fluid have also been linked to the pain associated with fibromyalgia (Bjersing and others 2013).

Beyond the scope of pain pathology, microRNAs represent a viable method of treatment. Single microRNA sequences can modify the expression of multiple genes, which is beneficial in treating complex conditions such as chronic pain that involve gene expression modifications across multiple tissues and genes. For example, when microRNA-124 was up-regulated in female mice with spared nerve injury–induced neuropathic pain, their pain response was normalized and their mechanical allodynia inhibited (Willemen and others 2012). Despite this, there are currently no microRNA interventions that have progressed to large-scale clinical trials focused on chronic pain (López-González and others 2017). Nevertheless, future studies should continue to explore this avenue, as epigenetics and early life trauma likely play a significant role in the onset and development of chronic pain during adolescence.

## Prophylactic Treatments for Chronic Pain

There are currently numerous analgesics available for chronic pain. These include medications such as opioids, antidepressants, nonsteroidal anti-inflammatory drugs, and other anti-inflammatory drugs. While many of these have demonstrated efficacy for pain symptoms in the short term (Ballantyne and Shin 2008; Payne 2000), they often lead to adverse reactions and tolerance, both of which reduce their long-term feasibility (Dumas and Pollack 2008). As such, increased efficacy and treatment options are required to address chronic pain symptoms.

There are currently very few pharmacologic agents that target the deficits and impairments associated with in utero trauma and ACEs. One treatment worth investigating, however, is oxytocin. Colloquially known as the “love hormone,” oxytocin is a neuropeptide produced by the posterior pituitary hypothalamus (Blanks and Thornton 2003). Oxytocin is a key hormone in parturition and lactation (Blanks and Thornton 2003; World Health Organization 2009) that has more recently been linked to prosocial behaviors and socialization (Marsh and others 2021). Oxytocin is commonly used as a pharmacologic agent during pregnancy to assist with increasing uterine contractions resulting in quicker labor processes (Bugg and others 2013). The hormone has also been implicated in metabolic and homeostatic mechanisms, suggesting that use of oxytocin may interfere with appetite and weight (McCormack and others 2020). Although the extent of oxytocin’s effects in the brain and systemically are still poorly understood (Erdozain and Peñagarikano 2020), recent research suggests that it has promise as an effective therapeutic for many neurologic conditions (for review, see Cochran and others 2014). Although the patterns of oxytocin levels during pregnancy are still contentious, studies have demonstrated increases in oxytocin originating in the first trimester (Prevost and others 2014), within late pregnancy (Uvnäs-Moberg and others 2019), and during labor (Leake and others 1981). Moreover, levels within humans range quite significantly (Dawood and others 1979; Levine and others 2007), and there is currently no consensus on what a normal oxytocin level is. Evidence suggests that increases in oxytocin positively correlate to maternal-infant bonding quality (Kohlhoff and others 2017). A systematic review determined that mothers with more secure styles of parenting had higher levels of oxytocin when compared with mothers with a more insecure attachment style (Galbally and others 2011). The authors theorized that higher oxytocin levels provide increased interactions with the dopaminergic reward pathways, leading to a positive affective state when bonding with the infant. Rodent models, however, show that oxytocin levels and oxytocin receptor levels are linked to maternal care, as measured by observable licking and grooming behaviors (Francis and others 2000; Pedersen and Prange 1979). Oxytocin is highly conserved between rodents and humans, but the distribution of oxytocin receptors throughout the CNS may be species specific, which corresponds to differences in social and sexual behavior (Lim and Lajtha 2006).

**Figure 5.**
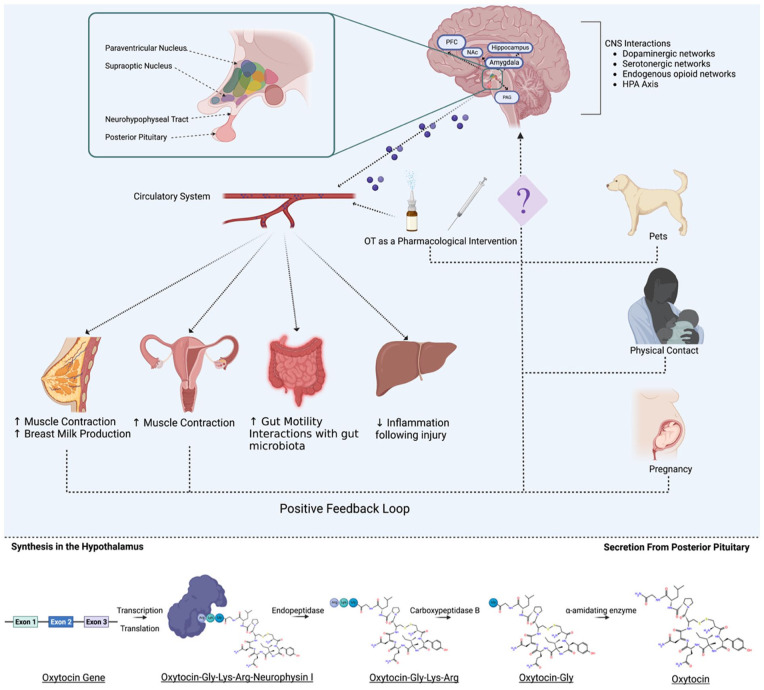
Oxytocin function and downstream effects. Oxytocin is synthesized within the hypothalamus and is projected to other regions of the brain or travels to the posterior pituitary gland where it is secreted to the periphery via the circulatory system. It exerts effects on mammary glands, uterine contractions, gut motility, and renal inflammation. In the case of uterine contractions and breast milk production, these functions can induce a positive feedback loop resulting in increased oxytocin production. Pregnancy induces an increase in oxytocin production, as do environmental factors such as physical touch, pets, or pharmacologic intervention.

Importantly for maternal trauma and ACEs, oxytocin is believed to regulate maternal-infant bonds (Galbally and others 2011) and is capable of modulating gene expression systemically and within the brain. See Figure 5 for overview of oxytocin generation and transmission. Although not examined in the context of early adversity, oxytocin treatment modified expression of the *GR* and mineralcorticoid (*MR*) genes in the hippocampus (Petersson and Uvnäs-Moberg 2003), as well as the epigenetic regulation of adipose- and angiogentic-related genes in adult rats (Eckertova and others 2011). Moreover, oxytocin levels in infancy are dynamic and can be epigenetically regulated by maternal care. High maternal engagement has been associated with increased expression of oxytocin in the offspring through changes in methylation of the *OXTR* gene (Krol, Moulder, and others 2019; Perkeybile and others 2019). Conversely, in adult clinical populations, acutely stressful events were capable of modifying methylation patterns of the *OXTR* gene (Unternaehrer and others 2012) with methylation status of the *OXTR* gene mediating the relationship between adversity (unmet material needs and neighborhood dysfunction) and depression (Simons and others 2017). The same is true for infants, where increased methylation of the *OXTR* gene was associated with enhanced activation of the inferior frontal cortex to fearful and angry stimuli but reduced activation of the same brain area in response to happy stimuli (Krol, Puglia, and others 2019). These results suggest that the oxytocin system is extremely plastic and susceptible to modification. Given that increases in methylation of the *OXTR* gene have been linked to a variety of neurologic conditions, such as autism spectrum disorder, anorexia nervosa, obsessive-compulsive disorder, depression, and anxiety (for review, see Kraaijenvanger and others 2019), it is plausible that early life manipulations, such as oxytocin administration, targeted toward decreased methylation of the *OXTR* gene could have beneficial effects, reducing an individual’s risk for the development of chronic pain. It is important to note, though, that oxytocin is believed to facilitate negative social emotions, including aggression, anger (Kemp and Guastella 2011), and withdrawal, and should therefore be explored in the context of perinatal trauma from a cautionary perspective.

Another potential therapeutic to improve the deficits associated with early adversity may be probiotics. Given that many “brain-centric” therapeutics have failed to adequately improve chronic pain outcomes, there is a need to expand on traditional strategies. Similar to oxytocin, probiotics target systemic processes and may therefore provide greater benefit for chronic pain symptomology associated with perinatal trauma. Probiotics are consumable forms of live bacteria that are hypothesized to be beneficial for human health. They can occur naturally in foods or be taken as supplements, and they are consumed to benefit gastrointestinal health. However, research conducted within the last decade has demonstrated that bacteria can exercise significant influence on other regions within the body and, in particular, play an important role in regulation of CNS functions (Cryan and others 2019). The gut microbiota and the brain have bidirectional communication, known as the *gut-brain axis*, which has been shown to affect white matter architecture (Ong and others 2018), microglial phenotypes (Wang and others 2018), and neurotransmitter activity (González-Arancibia and others 2019; Reigstad and others 2015; Strandwitz 2018). Rodent models have revealed that probiotic administration induces improved maternal behaviors (O’Leary 2019). Yet, these results have yet to be replicated in human populations (Rutten and others 2016). Probiotics have had a beneficial impact on offspring, alleviating anxiety- and depression-related symptoms (Liu and others 2019), which are common long-term consequences associated with ACEs (Lee and others 2020). Administration of *Bifidobacterium infantis* in rodents exposed to maternal separation increased pain thresholds (McKernan and others 2010) and stabilized noradrenaline levels within the brain (Desbonnet and others 2010). Although this study did not investigate any outcomes related to chronic pain development, it did confirm alleviation of depressive-like behavior induced by ACEs, which is a common comorbidity associated with chronic pain.

There have been multiple studies investigating pain and nociceptive behaviors in rodents exposed to gut microbiome manipulations (Amaral and others 2008; Verdú and others 2006). For example, inflammatory-induced nociception was reduced in mice lacking a gut microbiome, suggesting that the microbiota are important in the development of the nociceptive response (Amaral and others 2008). Similarly altered gut microbiome profiles resulted in decreases to neuropeptides such as substance P (Verdú and others 2006), which are known to modify nociceptive perception within the CNS. Administration of *Lactobacillus acidophilus* increased expression of multiple receptor types within the gut, including μ-opioid and cannabinoid receptors, and regulated analgesic responses in a rodent model (Rousseaux and others 2007). Although the neurobiological mechanisms are not fully elucidated, epigenetic factors are potentially involved. Microbiota have been demonstrated to modulate epigenetic mechanisms in models of depression (Zalar and others 2018). Few studies have focused on these factors in pain pathology, but it has been established that they influence neurologic processes. Within chronic pain models, there is existing research on probiotic therapies in irritable bowel syndrome or similar conditions and their associated pain (Roman and others 2018). More widespread chronic pain profiles are yet to be investigated. However, the current evidence does suggest that bacteria are able to influence CNS outcomes (Ghaisas and others 2016). As such, probiotics represent a potential and relatively safe therapeutic that may alleviate symptoms associated with ACEs, as well as CNS changes associated with chronic pain.

## Conclusion

Chronic pain is a highly burdensome disease that significantly reduces quality of life. It is becomingly increasingly evident that early life trauma, pre- and postnatal, influences molecular mechanisms, namely epigenetic regulation, which may lead to large-scale cortical reorganization that underlies the chronification of pain. These epigenetic mechanisms may provide an effective mechanism to improve chronic pain management while targeting the comorbid symptoms. Understanding how these factors combine and interact is vital to providing individualized care and ultimately reducing the detrimental role that chronic pain plays in the lives of millions of people. Personalized medicine, tailored to not only the epigenome but also the environmental conditions of an individual, would be a substantial advancement in clinical care. Moreover, given the significant evidence demonstrating sex differences within epigenetic processes, neurodevelopment, and chronic pain prevalence, this remains an important area of research worthy of future consideration. Most important, well-designed and robust animal models can be used to better address chronic pain in the clinic, if we can improve our understanding of how genes and the perinatal environmental combine to influence neural architecture and connectivity.
